# Investigation of oncolytic effect of recombinant Newcastle disease virus in primary and metastatic oral melanoma

**DOI:** 10.1007/s12032-023-02002-z

**Published:** 2023-04-06

**Authors:** Supaporn Numpadit, Chiaki Ito, Takaaki Nakaya, Katsuro Hagiwara

**Affiliations:** 1 School of Veterinary Medicine, Rakuno Gakuen University, Ebetsu, Hokkaido 069-8501 Japan; 2 Department of Infectious Disease, Kyoto Prefectural University of Medicine, Kamigyo-ku Kajii-cho, Kawaramachi-Hirokoji, Kyoto-shi, 602-8566 Japan

**Keywords:** Malignant melanoma (MM), rNDV, GFP, cIFNγ, Oncolytic virotherapy

## Abstract

Malignant melanoma is aggressive cancer with a high rate of local invasiveness and metastasis. Currently, the treatment options for patients with advanced-stage and metastatic oral melanoma are limited. A promising treatment option is oncolytic viral therapy. This study aimed to evaluate novel therapies for malignant melanoma using a canine model. Oral melanoma, which frequently occurs in dogs is used as a model for human melanoma, was isolated and cultured and used for the evaluation of the tumor lytic effect induced by viral infection. We constructed a recombinant Newcastle disease virus (rNDV) that promotes the extracellular release of IFNγ from the virus-infected melanoma. The expression of oncolytic and apoptosis-related genes, the immune response by lymphocytes, and IFNγ expression were evaluated in virus-infected melanoma cells. The results showed that the rate of rNDV infection varied according to the isolated melanoma cells and the oncolytic effect differed between melanoma cells owing to the infectivity of the virus. The oncolytic effect tended to be greater for the IFNγ-expressing virus than for the GFP-expressing prototype virus. Additionally, lymphocytes co-cultured with the virus showed induced expression of Th1 cytokines. Therefore, recombinant NDV expressing IFNγ is expected to induce cellular immunity and oncolytic activity. This oncolytic treatment shows promise as a therapeutic approach for melanoma treatment once evaluated using clinical samples from humans.

**Supplementary Information:**

The online version contains supplementary material available at 10.1007/s12032-023-02002-z.

## Introduction

Malignant melanoma occurs in various parts of the human body, but the most common sites are the hand and feet, followed by the head and neck, trunk, and mucosa [1]. According to the classic Clark classification, the four types of melanomas are lentigo lentiginous melanoma (ALM), nodular melanoma (NM), superficial spreading melanoma (SSM), and lentigo malignant melanoma (LMM), while those that occur in the mucosa are classified as mucosal melanoma (MCM). Some cannot be classified into any of these categories. In the United States, SSM accounts for 70%, ALM is < 5%, and MCM is very rare [2, 3]. However, in Japan, ALM (40%) and MCM (10%) account for half of the cases [1].

In humans, oral melanoma is aggressive malignancy and has a worse prognosis than cutaneous melanoma [4]. Oral melanoma accounts for 0.2% to 8% of all melanomas [5] and 1% to 2% of all oral malignancies [6]. Currently, melanoma treatment in humans includes surgical excision, radiation therapy, chemotherapy, melanoma vaccines, immune therapy, and targeted therapy [7]. However, advanced-stage melanoma and metastasis are difficult to treat. Therefore, it is necessary to find a therapy to increase the survival rate and quality of life for patients with advanced-stage disease.

In a report comparing postoperative adjuvant therapy with recurrence-free survival (RFS) rates performed for one year, the RFS rates in the COMBI-AD (dabrafenib plus trametinib), CheckMate 238 (nivolumab), and EORTC 1325 (pembrolizumab) were 88%, 70%, and 75%, respectively. Dabrafenib plus trametinib, a molecularly targeted therapy, is more effective. However, at the end of treatment, this difference disappears, with 3-year RFS rates of 58%, 58%, and 63.7%, respectively [8]. These results indicate that the tendency to relapse from the moment of discontinuation of its administration is a common problem with molecularly targeted drugs. In malignant melanoma, the number of genetic mutations (TMB) varies by disease type and is high in SSM but very low in ALM and MCM [9]. For this reason, some have suggested that immune checkpoint inhibitors (ICIs) may be more effective in Europe and the United States, where SSM is more common, but in Japan, where ALM and MCM account for half of all patients, ICI may be less effective. Among ICI, the frequency of immune-related adverse events (irAE) does not change much with increasing doses of anti-PD-1 antibody as a single agent, but the frequency of adverse events (AEs) (e.g., gastrointestinal disorders) increases with the dose of anti-CTLA-4 antibody, both as a single agent and in combination [10]. Furthermore, when anti-PD-1 and anti-CTLA-4 antibodies are combined, various irAEs occur simultaneously, and gastrointestinal symptoms, skin and liver damage are more likely to occur when they are combined simultaneously [11].

Considering the above, we investigate the use of oncolytic viruses as adjuvant therapy in patients with melanoma. In this study, we propose that the use of dogs is useful as a clinical model for melanoma therapy with oncolytic viruses because metastases from primary tumors occur frequently and in a relatively short period (within a year) after surgery, similar to those in humans.

Melanoma is common cancer in canines. The most common location was hairy skin. However, primary melanomas can occur in the oral cavity, gastrointestinal tract, footpad, eye, nail bed, nasal cavity, anal sac, or mucocutaneous junction [12]. Oral cancer accounts for approximately 6% of canine cancer [12]. Malignant melanoma is the most common type of canine oral tumor and is reported to be aggressive cancer with high local invasiveness and a high rate of metastasis [13]. Melanomas can occur in the oral cavities of dogs along the gingiva, lips, tongue, and hard palate [14]. Currently, treatment options for malignant melanoma include surgery, radiation therapy, and immunotherapy in the veterinary field. Surgical excision is the first-choice treatment. However, the available treatment options for canines with advanced-stage disease are limited and the prognosis is very poor. Therefore, the identification of a promising new strategy for cancer therapy is important. One promising therapeutic approach is oncolytic virotherapy. Several oncolytic viruses have been used for canine cancer therapy [15]. In recent years, immunotherapy, including targeted antibodies, checkpoint inhibitors, cytokines, and adjuvants, have been applied as monotherapy or in combination with current treatments for human and dog cancer. Melanomas encountered in dogs are primary tumors and present metastases that recur postoperatively in a relatively short period compared to humans. Due to the relative ease with which these melanomas can be obtained, it is beneficial to use canine clinical samples to study human treatment models.

Oncolytic virotherapy has recently been approved as a novel therapeutic agent for cancer treatment. An oncolytic virus selectively infects, replicates, and kills cancer cells without harming normal cells [16]. In humans, the benefits of oncolytic viruses for the treatment of cancer have been studied [17]. Oncolytic viruses are being developed clinically as treatment options in humans. Herpes simplex virus (HSV) has been used to treat patients with cutaneous or subcutaneous metastases of the breast, epithelial cancer of the head and neck, gastrointestinal adenocarcinoma, and malignant melanoma [18]. Patients with metastatic melanoma are currently being treated with HSV in a phase II study [19]. An adenovirus formulation has been reported to be a treatment option for bladder cancer [20]. Reoviruses have been used for the treatment of prostate cancer [21], metastatic colorectal cancer [22] and malignant glioma [23]. Several oncolytic viruses have been studied in the veterinary field; however, only a few clinical trials have reported the use of oncolytic viruses for the treatment of canine cancer [15]. For example, strains of canine adenovirus, canine distemper virus, and vaccinia virus have been used for canine cancer therapy in preclinical studies. Further, adenovirus has been used for the treatment of canine osteosarcoma and canine mammary carcinoma [24]. The measles virus (MV) belongs to the genus *Morbillivirus* of the *Paramyxoviridae* family and has been reported to treat canine mammary cancer [25].

The Newcastle Disease Virus (NDV) is an avian paramyxovirus with a negative single-stranded RNA genome. It is characterized by selective viral replication in tumor cells [26]. NDV is an avian pathogen that causes respiratory diseases. However, this virus is an oncolytic virus that replicates tumors and is selective in mammals. NDV-infected tumor cells have been reported to produce type I interferons (IFNs), induce the expression of class I major histocompatibility complex (MHC) and cell adhesion molecules, for example, hemagglutinin-neuraminidase (HN) protein on the surface of tumor cells increases the expression of class I MHC [27]. NDV has also been proposed for the treatment of human carcinoma [28]. Phase I and II clinical trials are progressing to evaluate NDV for treatment in humans [29]. A recombinant NDV-green fluorescent protein (GFP) has also been developed and has been reported to exert anti-tumor activity and anti-tumor immunity-inducing effects using tumors derived from mice [30, 31]. Previously, we generated a canine IFNγ (cIFNγ) gene expressing recombinant NDV (rNDV) using reverse genetics. Virus-infected cells induced cell death without producing any infectious virus particles because the amino acid sequence at the viral F protein cleavage site was transformed (G-R-Q-G/S-R; L) [32]. Recombinant NDV (rNDV) is safer than wild-type NDV for patient inoculation. NDV-infected tumor cells have been reported to induce apoptosis through caspase activation [33]. The indirect oncolytic mechanism is an immune-mediated response associated with both innate and adaptive immune responses [27, 34].

Interferon-γ (IFNγ) is the only member of the type II interferon family, which is generated following several innate and adaptive immune cell responses. IFNγ is a cytokine with antitumor activities, involved in the control of tumor initiation and progression [35]. IFNγ is useful for adjuvant immunotherapy in different types of cancer [36]. Interleukin-2 (IL-2) is a T cell growth factor. It is mainly secreted by CD4+ and CD8+ T lymphocytes and can stimulate proliferation of cytolytic activity and cytokine secretion by T lymphocytes and natural killer cells [37]. In humans, antitumor efficacy has been reported in patients with metastatic melanoma [38]. Interferon regulatory factor 1 (IRF-1) is a tumor suppressor gene with antiproliferative and pro-apoptotic effects on cancer cells [39]. Previous descriptions of IRF-1 expression have been associated with caspase activation [40].

The purpose of this study was to develop a novel therapy for malignant oral melanoma using the recombinant Newcastle disease virus. In this study, we applied the oncolytic recombinant NDV (rNDV) to induce cell-mediated immunity in treated patients and succeeded to achieve the oncolytic activity together with the production of canine IFNγ by rNDV-cIFNγ infection.

## Materials and methods

### Melanoma cell lines

Four canine melanoma cell lines (KMeC, LMeC, PU, and Mi) were used in this study. All cell lines were kindly provided by Dr. Kadosawa [41, 42]. Canine melanoma cell lines were cultured in RPMI-1640 medium (Sigma-Aldrich, Co., USA) medium containing antibiotics (200 U/mL penicillin Meiji, Tokyo, Japan) and 200 μg/mL streptomycin (Meiji, Tokyo, Japan), supplemented with 10% fetal bovine serum (FBS; Gibco, Waltham, MA, USA). The cells were maintained at 37 °C in a humidified atmosphere containing 5% CO_2_. All cell lines were maintained by passaging and used during logarithmic growth (Table 1).

**Table 1:** Origin of canine melanoma cell lines

Melanoma cell line	Dog breed	Age	Tumor origin	Morphologic cell type	WHO Stage	Clinical Stage	Reference
KMeC	Mongrel	14	Primary oral melanoma	Spindle cell	T3N1M0	4	Inoue et al., 2004 [41]
LMeC	Beagle	9	Metastatic mandibular lymph node of oral melanoma	Spindle cell	T4N1M0	4	Inoue et al., 2004 [41]
PU (CMM 1)	Toy poodle	12	Oral malignant melanoma	Spindle cell	T2bN1M0	3	Ohashi et al., 2001 [42]
Mi (CMM2)	Mongrel	13	Oral malignant melanoma	Spindle cell	T2aN0M0	2	Ohashi et al., 2001 [42]

### Recombinant virus

The expression of the canine interferon (IFN)γ gene was determined by RT-PCR amplification in PBMCs collected from healthy beagle dogs. To generate the recombinant virus, the green fluorescent protein (GFP) or canine IFNγ (cIFNγ) gene was inserted between the P-M region of the ORF genes of the Hitchner B1 strain NDV using reverse genetics. The amino acid sequence of the viral F protein cleavage site was transformed (G-R-Q-G/S-R; L). Exposure to the virus induced cell death only in infected cells, without producing any infectious virus particles. GFP-expressing rNDV or canine IFNγ (cIFNγ) were generated as previously described (Hagiwara et al., 2008). The virus was propagated in 9 or 10-day-old embryonated chicken eggs and transfected cells were injected into the allantoic cavities of embryonated chicken eggs. Infectious titers were determined from the number of cells infected with rNDV GFP or canine IFNγ (cIFNγ) by flow cytometry analysis (Beckman Coulter, Inc., United States) using mouse B16 cells. A total of 10^5^ B16 cells were infected with serial tenfold dilutions of rNDV-GFP or canine IFNγ (cIFNγ) in 24-well plates (four wells per dilution). The infected cells were incubated for 24 h before harvesting using 0.25% trypsin–EDTA (Sigma-Aldrich, St. Louis, MO, USA). The titer was determined using flow cytometry (Beckman Coulter, Inc., USA). The growth of both recombinant viruses was found to be greater than 10^7^/mL after 5 days of inoculation, and there was no change in virus growth in developing chicken eggs. The recombinant virus construction study was conducted with the approval of the Ethics Committee (Approval Number: 214).

### Detection of canine IFNγ

The production of cIFNγ in rNDV-cIFNγ- infected canine melanoma cells was examined by western blotting to confirm cIFNγ production. Oral melanoma cells (KMeC and LMeC, 5 × 10^5^ cells/well), were infected with rNDV-GFP and rNDV-IFNγ at an MOI of 2. The infected cells were harvested at 48 h post-infection (hpi), after which the cells were lysed with SDS sample buffer, and cIFNγ was detected by western blotting using anti-canine IFN-γ monoclonal antibodies (R&D Systems Inc., MN. USA), followed by reaction with anti-mouse IgG antibodies conjugated with peroxidase to develop antigen-bound antibodies.

### Evaluation of infectivity

Four canine melanoma cells (CMC) were evaluated for rNDV infectivity. Each melanoma cell was cultured in RPMI-1640 medium containing 5% fetal bovine serum (FBS; Gibco, Waltham, MA, USA) in 24-well plates and infected with rNDV-GFP at an MOI of 2. Thirty hours post-infection, the cells were washed with phosphate-buffered saline (PBS), harvested with 0.25% trypsin–EDTA (Sigma-Aldrich, Co., USA), and treated with 0.5% formalin-PBS. The cells were then subjected to flow cytometry (FCM); (Beckman Coulter, Inc., United States) to evaluate the infectivity of rNDV-GFP in canine melanoma cell lines.

### IFNγ response of canine PBMC by recombinant virus-infected cells

CMC (KMeC and LMeC cells), 5 × 10^5^ cells/well were cultured with RPMI-1640 containing 10% fetal bovine serum (FBS; Gibco, Waltham, MA, USA) in 24-well plates overnight, and then were infected with rNDV-GFP or rNDV-cIFNγ at an MOI of 2, and incubated for 30 h. To verify the biological activity of cIFNγ produced from the cIFNγ recombinant virus, IFNγ response was examined using PBMCs from healthy dogs. PBMCs were obtained from healthy 7–10-year-old beagle dogs. Blood samples were collected from the jugular veins of the five dogs. Blood was diluted with PBS and PBMCs were obtained by density gradient centrifugation using Ficoll solution (density of 1.077, Biocoll Separating Solution, Biochrom AG, Berlin, Germany). PBMCs (10^6^/mL) from healthy dogs were added to cells infected with rNDV in 24-well plates. PBMCs were collected and suspended in RLT buffer (QIAGEN GmbH Qiagen Strasse 1, 40724 Hilden, Germany) for RNA extraction using an RNA extraction kit (QIAGEN, RNeasy Kits, QIAGEN GmBH, Qiagen Strasse 1, 40724 Hilden, Germany), and performed according to the manufacturer’s protocol. The RNA samples were stored at − 80 °C until cDNA synthesis. For cDNA synthesis, ReverTra Ace™ qPCR RT Kit (TOYOBO Co., LTD, Japan) was used to synthesize high-quality cDNAs for real-time PCR, and samples were kept at -30 °C until the real-time PCR assay. The reverse transcriptase was then inactivated by heating it to 85 °C for 5 min. The expression of IFNγ-stimulated genes was examined by real-time PCR using the following primers: *IRF1*: F 5′-TGTGGTGGCTGCACTAAGAG-3′, R:5′-GGCAAGGTCCACAAGAAAAA-3′. The study was carried out with the approval of the Ethics Committee according to the Laboratory Animal Control Guidelines at Rakuno Gakuen University (Approval Number: VH21B9).

### Evaluation of cytotoxicity

To evaluate the cytotoxicity of the rNDVs, a cellular cytotoxicity assay was performed using the lactate dehydrogenase (LDH) Release Analysis Kit. (Cytotoxicity LDH Assay Kit-WST, Dojindo Molecular Technologies, Inc., Kumamoto, Japan). This kit was used to determine cytotoxicity by measuring the activity of LDH released from damaged cells.

The LDH assay kit WST (Dojindo Molecular Technologies, Inc., Japan) was used for the study. The 4 canine melanoma cells were cultured with RPMI-1640 containing 5% fetal bovine serum (FBS; Gibco, Waltham, MA, USA) in 96-well plates, infected with rNDV-GFP and rNDV-IFNγ at MOI of 2. LDH levels were tested at 48 h and 72 h after the virus infection. Lysis buffer (20 μL) as the mock condition was used as a high control. The plate was incubated at 37 °C for 30 min in a CO_2_ incubator. Next, 100 μL of the cell suspension was added to each well of a 96-well tissue culture plate in the new plate and 100 μL of assay medium was added to the plates. The plates were incubated at 37 °C for 30 min in a CO_2_ incubator. Next, 50 μL of stop solution was added to each plate. Absorbance was measured at 490 nm using a microplate reader (Ultramark™ Microplate Reader, BIO-RAD, USA), the average absorbance was calculated from each triplicate set of wells, and the background control value was subtracted from each absorbance. Cytotoxicity was calculated using the following equation:

Cytotoxicity (%) = (A−C)/(B−C) × 100 A: test substance, B: High control, C: Low control.

### *Caspase 3* gene expression in melanoma cells

KMeCs are cells isolated from primary oral melanoma, and LMeCs are lymph node metastatic cells. We compared Caspase 3 expression after viral infection of both cell lines to determine whether differences in their cytotoxicity to the virus are related to Caspase 3 expression. CMC (LMeC and KMeC) were cultured in RPMI-1640 containing 10% fetal bovine serum (FBS; Gibco, Waltham, MA, USA) in 24-well plates and then cells were infected with rNDV-GFP or rNDV-cIFNγ at an MOI of 2. The expression of the *caspase 3* gene was evaluated at 24, 48, and 72 h after infection. RNA extraction, cDNA synthesis, and real-time PCR were performed using the same protocols described above. The expression of *caspase 3* was examined by real-time PCR using the following primers: F-5′-GAAGATCATAGCAAAAGGAG-3′ and R:5′-TGTCTCAATGCCACAGTCCA-3′.

### Cytokine gene expression in dog PBMC

*IFNγ* and *IL2* were evaluated to assess Th1 response in PBMC in response to rNDV-infected melanoma. Healthy canine-derived PBMCs were co-cultured with rNDV-cIFN-γ-infected tumor cells. KMeC cells, 5 × 10^5^ cells/well were cultured with RPMI-1640 containing 10% FBS (Gibco, Waltham, MA, USA) in 24-well plates overnight, infected with rNDV-GFP and rNDV-IFNγ at an MOI of 2, and incubated for 30 h. Healthy canine PBMCs were then added to the rNDV-infected melanoma cells. PBMCs were collected 12 h after incubation and RNA was isolated using an RNeasy mini kit (Qiagen, Hilden, Germany). The expression of *IFNγ*, *IL-2,* and *GAPDH* was examined by real-time PCR using the following primers: *IFNγ* F:5′-GTTGCTGCCTACTTGGGAAC-3′, R:5′-GGCGTCTGACATGCCTCTA-3′; *IL-2* F:5′-CCTCAACTCCTGCCACAATGT-3′, R:5′-TGCGACAAGTACAAGCGTCAGT-3′; *GAPDH* F:5′-TCCCTCAAGATTGTCAGCAA-3′, R:5′-TGGATGACTTTGGCTAGAGGA-3′.

### Statistical analysis

Differences between two sample groups were calculated using Student’s *t *test. Multiple comparison analyses were performed using the Tukey–Kramer multiple comparison method. Values were considered statistically significant at *p* < 0.05.

## Results

### Detection of canine IFNγ by western blotting

**Fig. 1:**
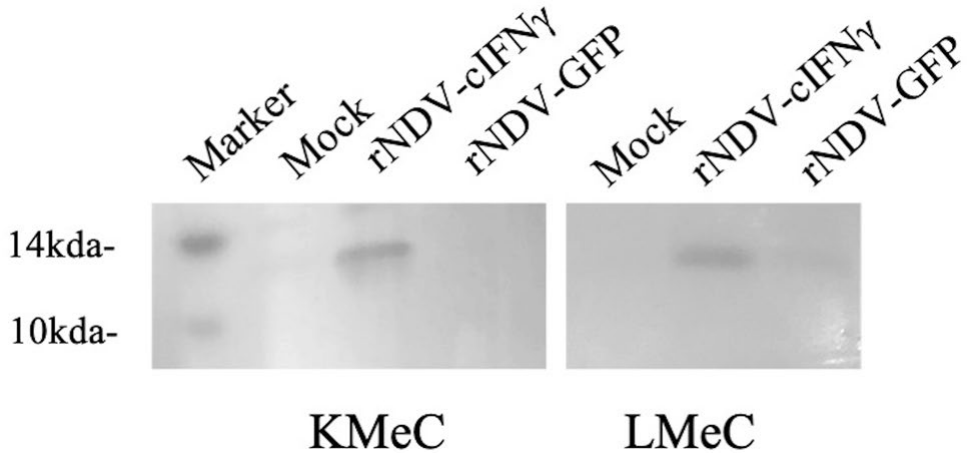
Detection of canine IFNγ in rNDV- IFNγ infected cells. Detection of canine IFNγ in melanoma cells KMeC and LMeC infected with rNDV-cIFNγ at an MOI of 2. Infected cells were harvested and lysed with SDS sample buffer at 48 hpi. Canine IFNγ was detected by western blotting analysis using anti-canine IFN-γ monoclonal antibodies (R&D Systems Inc.. MN. USA)

The expression of canine IFNγ was examined by western blotting in rNDV-cIFNγ-infected melanoma cells, using anti-canine IFNγ antibodies (Fig. 1).

### The expression of canine PBMC in coculture with rNDV-cIFNγ infected tumor cells

**Fig. 2:**
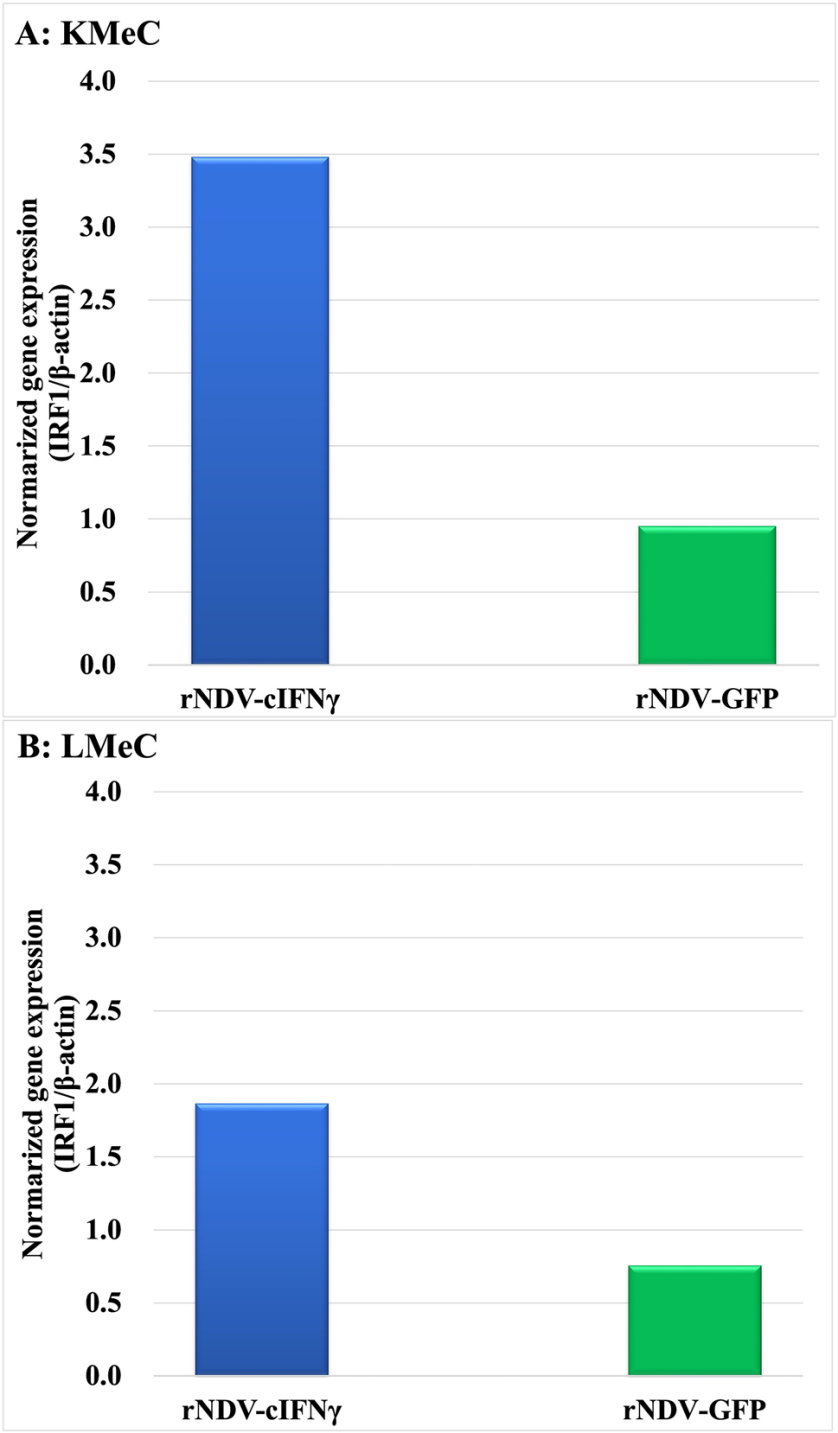
Gene expression of canine PBMC in coculture with rNDV-cIFNγ-infected tumor cells. **A** Higher IRF1 expression is observed when KMeC cells are infected with rNDV-cIFNγ. **B** Higher IRF1 expression is induced when LMeC cells are infected with rNDV-cIFNγ

To investigate IFNγ-induced gene expression in infected canine melanoma cells, we selected the KMeC and LMeC cells for this study. KMeC originates from primary oral melanoma and LMeC originates from the metastatic mandibular lymph node of oral melanoma. Healthy canine-derived PBMCs were co-cultured with rNDV-cIFN-γ-infected tumor cells infected with rNDV-cIFN- (KMeC or LMeC) for 48 h. IFNγ-induced gene expression IRF1 in PBMC was analyzed using real-time PCR. The results of duplicate experiments are shown by normalizing gene expression with β-actin and setting MOCK expression to 1. The results showed that 48 hpi, KMeCs, or LMeCs infected with rNDV-cIFNγ tended to have higher IRF1 expression of IRF1 than those infected with rNDV-GFP (Fig. 2).

### Evaluation of the infectivity of recombinant NDV in canine melanoma cell lines.

**Fig. 3:**
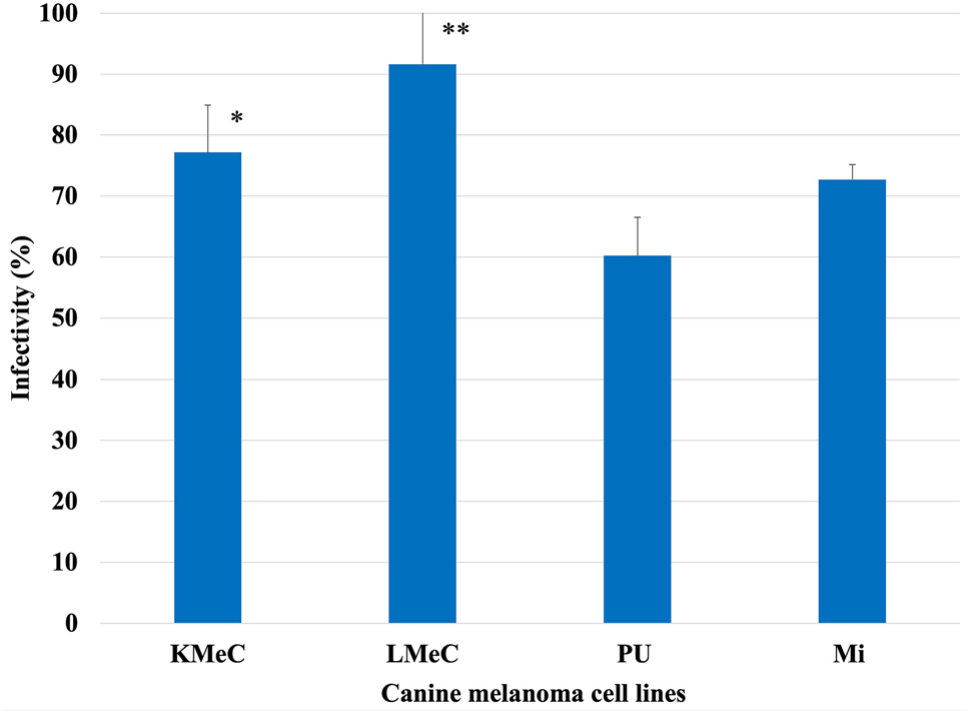
Infectivity of rNDV-GFP in canine melanoma cell line. Infectivity of rNDV-GFP in oral melanoma cell lines. LMeC and KMeC cells had a significantly higher infection rate than PU cells. (**P < 0.01, *P < 0.05). The average infectivity of rNDV in canine melanoma cells was 92–60%

To investigate the infectivity of rNDV in canine melanoma cell lines, tumor cells were infected with rNDV-GFP and the rNDV infection rate was calculated from the ratio of GFP expression cells by FCM analysis. rNDV infection rates in KMeC, LMeC, PU, and Mi cells were 77%, 92%, 60%, and 73%, respectively. The results showed differences in infectivity between melanoma cell lines. Therefore, factors affecting rNDV were examined in canine melanoma cell lines (Fig. 3).

## Cytotoxicity of rNDV-infected melanoma

**Fig. 4:**
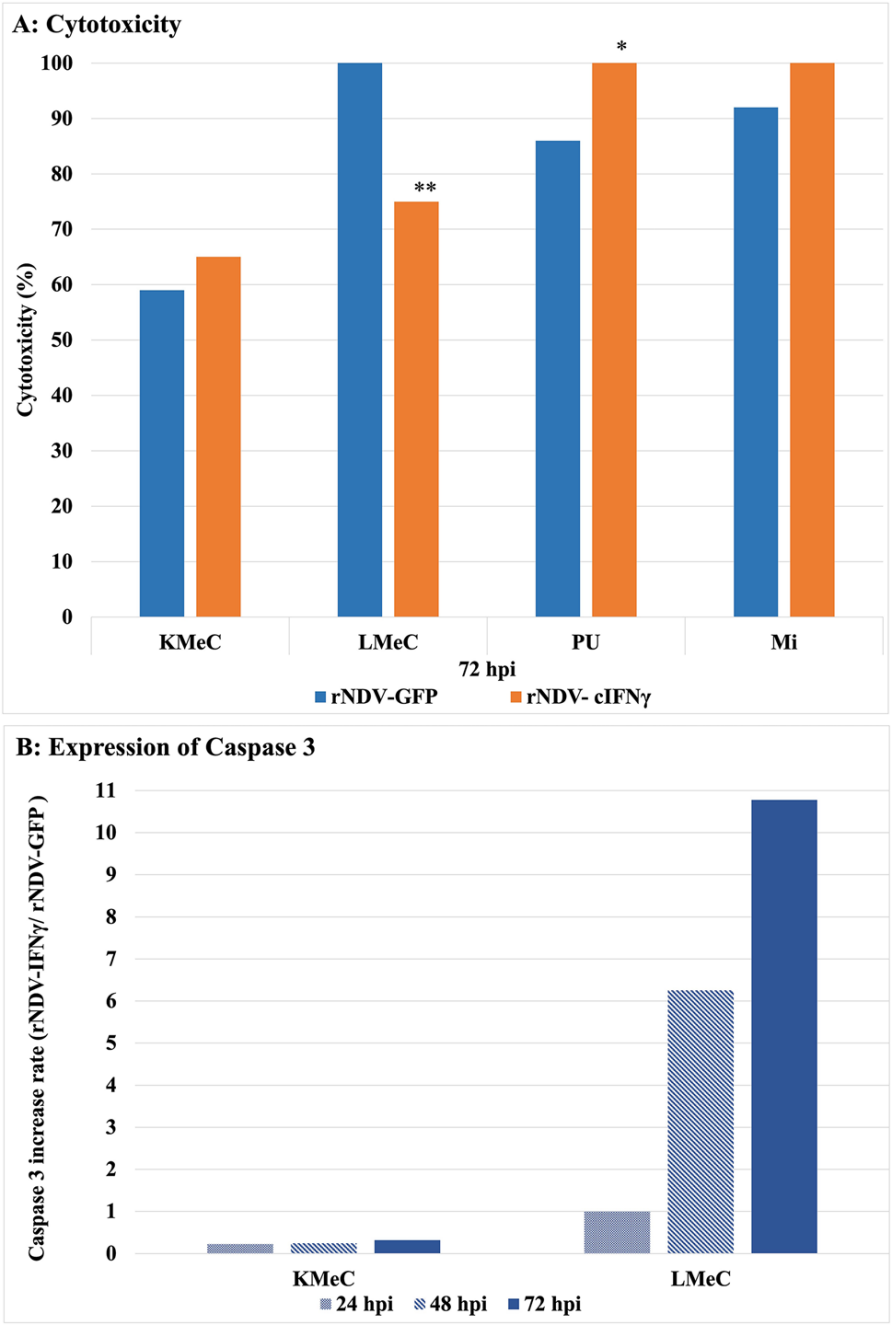
Cytotoxicity of rNDV-GFP and rNDV-IFN in canine melanoma cell line and caspase 3 increased rate in the melanoma infected with rNDVs. **A** The cytotoxicity of the canine melanoma cell line was evaluated at 72 hpi using an LDH assay. The cytotoxicity rates of LMeC and PU were significantly different (**p < 0.01, *p < 0.05) between rNDV-GFP and rNDV-IFNγ. **B** The expression of Caspase 3 in the cells infected with rNDV-cIFNγ vs. rNDV-GFP at 24, 48, and 72 hpi

To investigate cytotoxicity in canine melanoma cell lines. Tumor cells were infected with rNDV-GFP and -IFNγ at an MOI of 2. The LDH assay kit WST was used to evaluate cytotoxicity 48 and 72 h after viral infection. The rate of cytotoxicity of canine melanoma was dependent on the melanoma cell. Cytotoxicity induced in canine melanoma cells after rNDV-GFP and rNDV-IFNγ infection was as follows: KMeC (59%, 65%), LMeC (100%, 75%), PU (86%, 100%), and Mi (92%, 100%), respectively. (Fig. 4A). Differences in cytotoxicity between types of melanoma cell lines and the levels of cytotoxicity for canine melanoma were greater in rNDV-IFNγ-infected cells, with the exception of LMeC.

### The expression of *Caspase 3* in the canine melanoma cell lines.

To investigate apoptosis-related gene expression in canine melanoma cells. CMCs (KMeC, LMeC, 1 × 10^5^ cell/well were infected with rNDV-GFP and rNDV-IFNγ at an MOI of 2. At 24, 48, and 72 hpi expression of caspase 3 was evaluated by real-time PCR. The expression of caspase 3 was significantly increased by active caspase 3 at 48 and 72 hpi when infected with rNDV-IFNγ. In KMeC melanomas, IFNγ did not significantly upregulate caspase-3 expression, which was correlated with KMeC cytotoxicity, suggesting that there may be factors that affect caspase-3 expression as a characteristic of KMeC. Furthermore, increased levels of levels of Caspase 3 expression were observed in metastatic cells of LMeC and KMeC lymph node metastatic cells, indicating that metastasis of cells of the same origin may result in different expression of Caspase3 in response to the virus (Fig. 4B).

### Expression of *IFNγ and IL2* in canine PBMCs

**Fig. 5:**
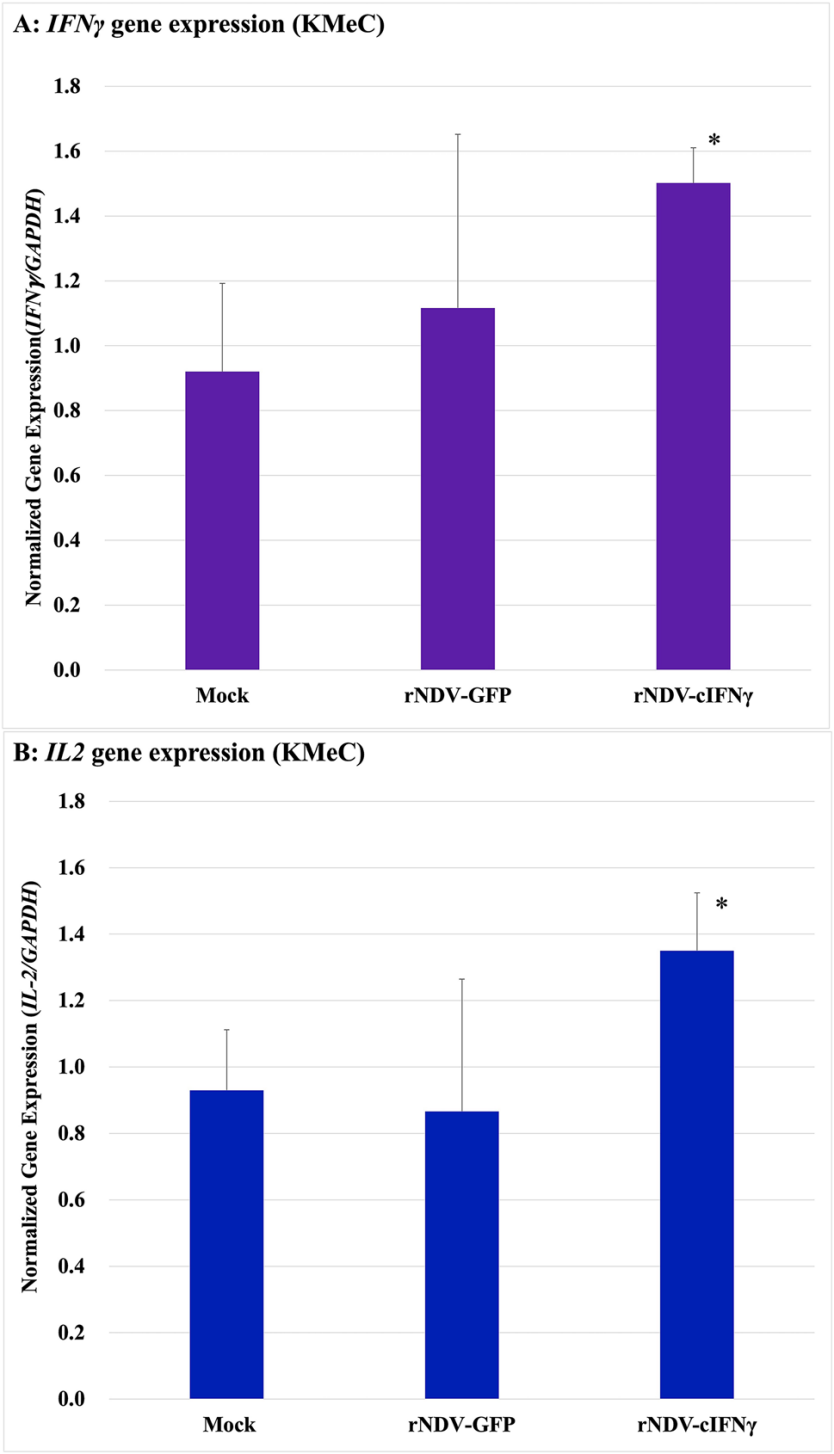
Detection of *IFNγ* and *IL2* gene expression. **A** KMeC have higher *IFNγ* levels, which were significantly different (*p < 0.05) from that between mock and rNDV-cIFNγ. **B** KMeC have significantly increased *IL-2* expression when compared to the rNDV-GFP and rNDV-cIFNγ groups, and shows significance (*p < 0.05, between rNDV-GFP and rNDV-cIFNγ)

*IFNγ* and *IL2* expression in PBMC of healthy dogs in response to rNDV-infected melanoma cells. *IFNγ* and *IL2,* which induces cellular immunity, tend to be higher in cells infected with rNDV-cIFNγ (Fig. 5).

## Discussion

In humans and canines, oral melanoma is aggressive and has a worse prognosis than cutaneous melanoma. In this study, we focused on the treatment of oral melanoma treated with oncolytic virus in vitro. We succeeded in creating an rNDV that produced canine (c) IFNγ in infected cells. The virus could easily be multiplied in chicken eggs to titers of approximately 10^7^ FFU/mL, which was similar to that of GFP-expressing rNDV. We performed an in vitro evaluation of the efficacy of the oncolytic virus using various canine melanoma cell lines. Canine melanoma cell lines (CMCs) were infected with rNDV-GFP or rNDV-cIFN-γ. The KMeC, LMeC, and Mi melanoma cells achieved rNDV-GFP infection rates of more than 70%, but PU showed an infection rate of 60%. The infection rate varied depending on the type of canine melanoma cell line used. Some cells with primary oral melanoma have been inferred to have a lower rate of viral infection. It has also been suggested that these properties may be different from those of cells derived from metastatic tumors. The tumor cells used in this study were derived from primary and metastatic tumors, suggesting that the origin of the tumor may be related to the oncolytic effect. This may be related to resistance to apoptosis by rNDV infection, but further research remains necessary to understand these phenomena. The infectivity of the virus depends on tumor origin and the degree of expression of the interferon-stimulated gene (ISG) in tumor cells. In a previous study, we described ISG in tumor cells [31]. Using KMeC and LMeC cells, we studied IRF1 expression in rNDV-infected cells. The results showed that the expression of IRF genes increased in lymphocytes co-cultured with cells infected with rNDV-cIFNγ, which suggested that cIFNγ produced after recombinant virus infection acts on surrounding lymphocytes and promotes the expression of IFN-related genes.

The cytotoxicity of canine melanoma cell was different among the canine melanoma cells, due to differences in the infectivity of the virus. The infectivity of the virus was dependent on tumor origin and the degree of expression of ISGs in tumor cells. Furthermore, the cytotoxicity differed between melanoma cells, leaving the task of clarifying their characteristics. Except for PU cells, the infection rate of rNDV exceeded 70%. However, despite the low infection rate in PU at 30 h post-virus infection, the cytotoxicity rate was high, suggesting that the rNDV infection of PU cells progressed by 72 h, resulting in high cytotoxicity. The results indicated that the cytotoxicity of the primary oral tumor origin in the rNDV-cIFNγ group was higher than that in the rNDV-GFP group. Conversely, IFNγ production was confirmed in cells infected with rNDV-cIFNγ infected cells and induced PBMC stimulation. Comparison of caspase 3 induction by different recombinant viruses showed this comparison was made as late as 72 h after viral infection, suggesting that where the actual cytotoxicity was higher, Caspase 3-induced apoptosis occurred within 72 h. The low cytotoxicity of KMeC may be related to induction of apoptosis, suggesting that there is some regulation of Caspase 3 activation after viral infection and induction of cell death. This phenomenon remains to be investigated in detail in viral therapy for melanoma. The possibility that cellular damage caused by viral infection may be affected by intracellular gene regulation in the tumor should also be considered [33]. It has been suggested that virus-induced apoptosis differs between primary and metastatic tumors, which in turn affects apoptosis during viral infection.

Like other cancers, melanoma is caused by genetic and epigenetic changes [43]. Various factors, such as alterations in multiple genes, have been considered to be involved in the progression of melanoma [44], such as genetic alteration in multiple genes.

Type 1 T helper cells (Th1) produce IFNγ and interleukin (IL) 2 which activate macrophages and are responsible for cell-mediated immunity and phagocyte-dependent protective responses. In this study, we determined the molecular viral components that induce adaptive and innate immune responses in canine PBMCs. KMeC cells are primary oral melanomas that originate in the gingiva. The expression of *IFNγ* and *IL-2* in PBMC co-cultured with KMeC infected with rNDV-cIFNγ was higher than in infection with rNDV-GFP. These findings suggest that lymphocytes co-cultured with rNDV-cIFN-γ-infected melanoma cells were Th1-induced. KMeC cells have a lower cytotoxicity rate for rNDV infection than metastasis-derived cells, suggesting that they may be resistant to cytotoxicity other than viral infections. However, other oral melanomas and melanoma cells metastasized from oral melanoma are similarly cytotoxic to rNDV infection, suggesting that some primary melanoma cells are resistant to viral infection-caused apoptosis. Since viral lysis in tumors can vary depending on tumor characteristics, even in the same melanoma, detailed studies on resistance to apoptosis may be necessary.

The recombinant NDV used in this study can infect melanoma cells, but the virus cannot multiply in melanoma cells due to a mutation in the F protein gene, and the virus infects the cells only once. In other words, only the administered virus infects tumor cells and causes cytotoxicity, which is expected to reduce side effects caused by virus multiplication [32, 45]. On the other hand, there is concern that repeated administration of the virus may reduce viral infection due to the antiviral effect of the host's immune response, but direct injection of the virus into the tumor mass is expected to ensure local infectivity. This point needs to be further investigated in future in vivo studies.

## Conclusion

We successfully produced oncolytic viral construct (rNDV-cIFNγ) that infects melanoma cells. This virus was highly infectious to primary and metastatic melanoma and similar to the prototype rNDV-GFP, showed > 70% cytotoxicity against all but a subset of primary tumors. IFNγ produced by rNDV-infected melanoma showed induction of Th1 cytokines in co-cultured lymphocytes and IFNγ was also induced by rNDV-GFP. In conclusion, rNDV-cIFNγ induces cytotoxicity in melanoma cells and Th1 cells in surrounding lymphocytes, which is expected to induce cellular immunity and tumor lysis. Its high cytotoxicity against metastatic tumors is also expected to find application as an adjuvant therapy to inhibit metastasis and improve postoperative quality of life. In the future, it will be necessary to verify the antitumor and metastatic inhibitory effects in vivo*.*

## Supplementary Information

Below is the link to the electronic supplementary material.

Supplementary file1 (DOCX 11002 kb)—** Supplementary Fig. 1** Microscopic observation of Canine Melanoma cells. KMeC (primary oral melanoma), LMeC (metastatic of the mandibular lymph node), PU (oral malignant melanoma), and Mi (oral malignant melanoma) are shown. Microscopic findings of each melanoma cell line are spindle-shaped, and nucleoli are observed (x200). **Supplementary Fig. 2** Construct of rNDV-GFP or -canine IFNγ genome. pNDV/B1, containing the full-length cDNA of the Hitchner B1 strain, was constructed, and additional restriction enzyme sites (XbaI, nt 3163–3168) were created as genetic tag sequences. We chose the newly introduced XbaI site, located between the P and M genes, to insert the canine IFNγ or green fluorescent protein (GFP) gene.

## Data Availability

The datasets generated during and/or analyzed during the current study are available from the corresponding author on reasonable request.

## Abbreviations

ALM: Acral lentiginous melanoma
cIFNγ: Canine interferon gamma
FCM: Flow cytometry
GFP: Green Fluorescent protein
Hpi: Hours post-infection
HN: Hemagglutinin-neuraminidase
IFNγ: Interferon gamma
IL-2: Interleukin 2
ICIs: Immune checkpoint inhibitors
ISGs: Interferon-stimulated gene
IRF-1: Interferon regulatory factor 1
LMM: Lentigo maligna melanoma
LDH: Lactate dehydrogenase
MM: Malignant melanoma
MCM: Mucosal melanoma
MHC: Major histocompatibility complex
NM: Nodular melanoma
NDV: Newcastle disease virus
PBMCs: Peripheral blood mononuclear cells
PBS: Phosphate buffered saline
QOL: Quality of life
RFS: Recurrence-free survival
rNDV: Recombinant Newcastle disease virus
SSM: Superficial spreading melanoma

## Declarations

### Research involving human and/or animal participants

Ethics Committee review and approval were obtained for research using PBMCs derived from animals (dogs). No human or clinical sample was used in this study. Recombinant virus construction experiments were conducted in accordance with the guidelines of the Japanese Ministry of Education, Culture, Sports, Science, and Technology and with the approval of the Ethics Committee.
