# Supplementary figures and images for: Investigation of oncolytic effect of recombinant Newcastle disease virus in primary and metastatic oral melanoma
Source: Med Oncol. 2023 Apr 6;40(5):138. doi: 10.1007/s12032-023-02002-z (PMC10079733; doi:10.1007/s12032-023-02002-z)

**Supplementary Figure 1**

**
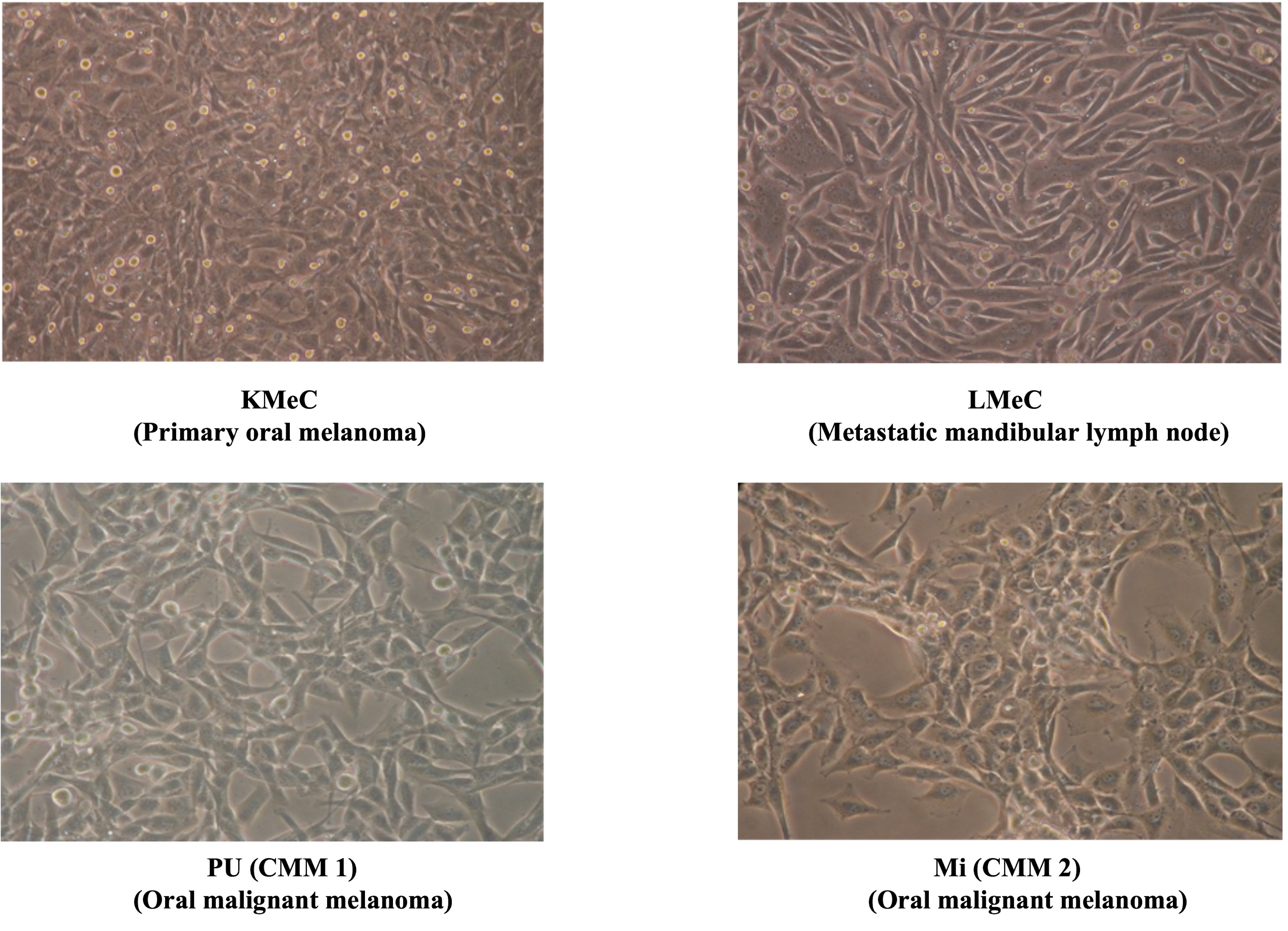
**

**Supplementary Fig. 2**


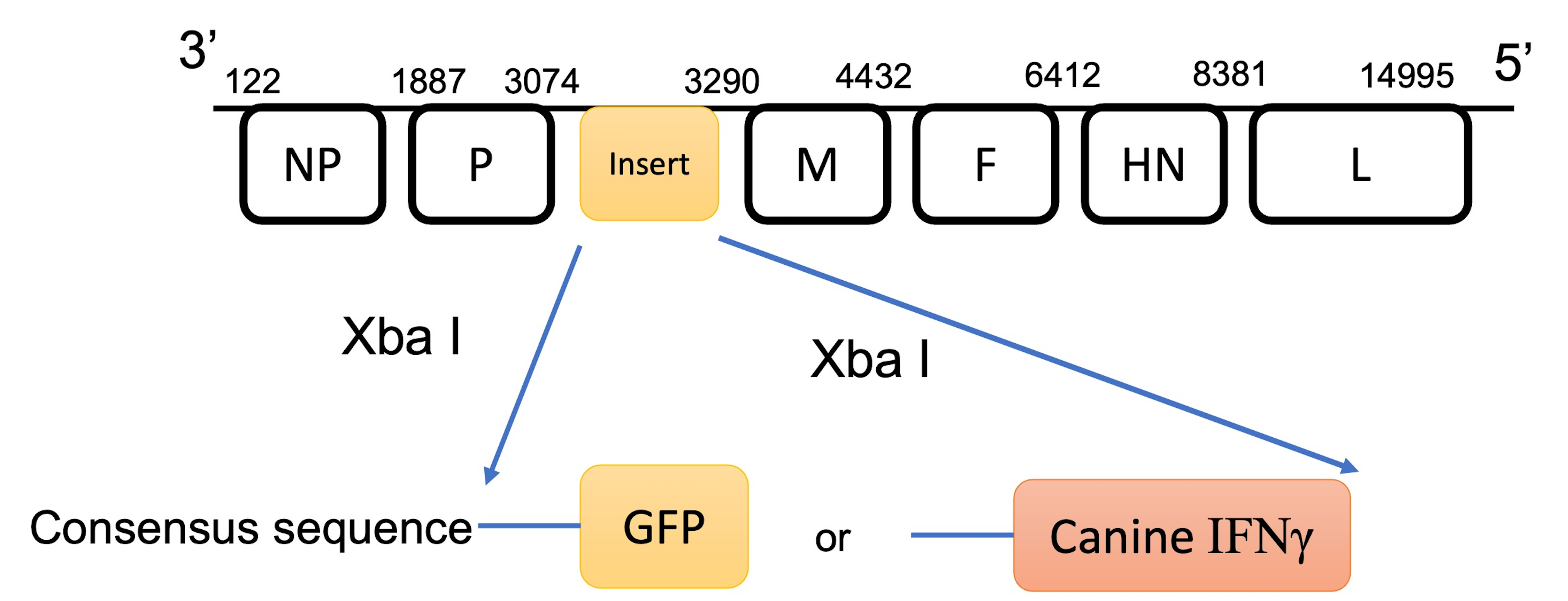

Supplement: Supplementary file 1 — Supplementary file1 (DOCX 11002 kb)— Supplementary Fig. 1 Microscopic observation of Canine Melanoma cells. KMeC (primary oral melanoma), LMeC (metastatic of the mandibular lymph node), PU (oral malignant melanoma), and Mi (oral malignant melanoma) are shown. Microscopic findings of each melanoma cell line are spindle-shaped, and nucleoli are observed (x200). Supplementary Fig. 2 Construct of rNDV-GFP or -canine IFNγ genome. pNDV/B1, containing the full-length cDNA of the Hitchner B1 strain, was constructed, and additional restriction enzyme sites (XbaI, nt 3163–3168) were created as genetic tag sequences. We chose the newly introduced XbaI site, located between the P and M genes, to insert the canine IFNγ or green fluorescent protein (GFP) gene. [file 12032_2023_2002_MOESM1_ESM.docx]
